# Macrolide-Resistant *Mycoplasma pneumoniae* in Humans, Ontario, Canada, 2010–2011

**DOI:** 10.3201/eid1909.121466

**Published:** 2013-09

**Authors:** AliReza Eshaghi, Nader Memari, Patrick Tang, Romy Olsha, David J. Farrell, Donald E. Low, Jonathan B. Gubbay, Samir N. Patel

**Affiliations:** Ontario Agency for Health Protection and Promotion, Toronto, Ontario, Canada (A. Eshaghi, N. Memari, P. Tang, R. Olsha, D.J. Farrell, D.E. Low, J.B. Gubbay, S.N. Patel);; University of Toronto, Toronto (D.J. Farrell, D.E. Low, J.B. Gubbay, S.N. Patel);; Mount Sinai Hospital, Toronto (D.E. Low, J.B. Gubbay);; The Hospital for Sick Children, Toronto (J.B. Gubbay)

**Keywords:** macrolide resistance, community-acquired pneumonia, macrolides, Mycoplasma pneumoniae, bacteria, antibiotic resistance, antimicrobial resistance, Ontario, Canada

## Abstract

Antimicrobial drug resistance rates for *Mycoplasma pneumoniae* was determined in clinical specimens and isolates obtained during 2011–2012 in Ontario, Canada. Of 91 *M. pneumoniae* drug-resistant specimens, 11 (12.1%) carried nucleotide mutations associated with macrolide resistance in the 23S rRNA gene. None of the *M. pneumoniae* specimens were resistant to fluoroquinolones or tetracyclines.

*Mycoplasma pneumoniae* is a major cause of community-acquired pneumonia among children and adults (*1*). Macrolides are recommended for treatment of *M. pneumoniae* pneumonia (*1*).

High rates of macrolide-resistant *M. pneumoniae* have been reported in China (>90%) and Japan (87.1%) (*2**,**3*). In Europe, reports of macrolide resistance have ranged from 3% in Germany to 9.8% in France (*4**,**5*). In the United States, 8.2% of *M. pneumonia*–positive specimens identified during 2007–2010 were resistant to macrolides (*6*). *M. pneumoniae* confer macrolide resistance primarily as a result of nucleotide substitutions at specific positions in the V domain of the 23S rRNA gene. Mutations at nt 2063 (A2063T/G), 2064 (A2064G), and 2617 (C2617A/G) have been shown to be associated with increased MICs to macrolides, including erythromycin, azithromycin, and clarithromycin (*2**,**3**,**7**,**8*). Use of macrolides to treat macrolide-resistant *M. pneumoniae* result in lower effectiveness and increased clinical severity compared with macrolide-susceptible *M. pneumoniae (**9**)*. In contrast to macrolides, resistance to quinolones or tetracyclines among clinical isolates of *M. pneumoniae* has not been reported, although development of such resistance after use of increased concentrations of fluoroquinolones or doxycycline has been demonstrated in in vitro settings (*10**,**11*).

The Public Health Ontario Laboratory, which is the reference microbiology laboratory for the province of Ontario, provides molecular testing for detection of *M. pneumoniae* for hospitalized and ambulatory patients. In August 2011, the positivity rate for specimens with *M. pneumoniae* increased to 9.3% and peaked in December 2011 to 17.5%. During the same time, increased numbers of cases of *M. pneumoniae* were reported throughout Europe. In response to the increased positivity rate and lack of data for Canada on macrolide resistance in *M. pneumoniae*, we investigated antimicrobial drug susceptibility profiles of *M. pneumoniae* detected during February 2010–January 2012 by using molecular methods. In addition, available *M. pneumoniae* isolates were characterized by sequencing the P1 gene to determine the prevalence of circulating types in Ontario, Canada (*12**,**13*).

## The Study

During February 1, 2010–January 31, 2012, a total of 2,898 respiratory specimens were tested for *M. pneumoniae* and *Chlamydophila pneumoniae* by using a multiplex testing real-time assay (ProPneumo-1 Assay; Gen-Probe Inc., San Diego, CA, USA). A total of 96 specimens were positive for *M. pneumoniae*, and 16 specimens were positive for *C. pneumoniae.* Among *M. pneumoniae*–positive specimens, 67 (70%) and 29 (30%) were from the upper and lower respiratory tract, respectively. Six (6.0%) specimens were collected from children < 4 years of age, 48 (50%) from persons 5–20 years of age, 19 (20%) from persons 21–40 years of age, 19 (20%) from persons 41–60 years of age, and 23 (24%) from persons >65 years of age. All *M. pneumoniae*–PCR positive specimens were cultured and 42 (44%) of the 96 primary specimens yielded positive isolates.

Nested PCR amplification and DNA sequencing of the partial 23S rRNA gene were performed to detect mutations at nucleotide positions 2063, 2064, 2067, 2617 in the 23S rRNA gene, which are associated with macrolide resistance (*2**,**8*). In addition to macrolide resistance, molecular determinants of fluoroquinolones (*gyrA* and *parC*) and tetracycline (16S rRNA) resistance were also analyzed (*10**,**11*).

For macrolide resistance, 91 (95%) of 96 specimens were amplified and analyzed for mutations. Mutations that have been associated with macrolide resistance were found in 11 (12.1%) of the 91 specimens (Table 1). Of the 11 isolates with a mutant genotype, 10 (90.9%) contained a mutation at nucleotide position 2063 (A2063G), and 2 (18.2%) specimens had a mutation at position 2064 (A2064G). In 4 isolates, a mixed population of wild type and mutant at position 2063 were identified on sequence chromatograms. One specimen had wild type and co-mutations at positions 2063 and 2064. None of the specimens contained any mutations at positions A2067 or C2617.

**Table 1:** Macrolide-resistant *Mycoplasma pneumoniae* identified in Ontario, Canada, 2010–2011*

Patient	ID no.	Age, y/sex	Specimen collection date	Specimen source	Substitution in 23s rRNA			
					A2063	A2064	A2067	C2617
1	H72992–11	43/F	2011 Aug 8	SPT	A/G	A/G	A	C
2	C706158–11	10/M	2011 Aug 25	NP	A/G	A	A	C
3	K35611–11	38/F	2011 Sep 1	NP	G	A	A	C
4	C751048–11	44/F	2011 Sep 8	BAL	G	A	A	C
5	P54752–11	42/M	2011 Oct 6	NP	G	A	A	C
6	P54912–11	12/M	2011 Oct 13	NP	A/G	A	A	C
7	M29279–11	3/F	2011 Dec 9	NP	G	A	A	C
8	N223472–11	5/M	2011 Dec 14	BAL	G	A	A	C
9	N223473–11	5/M	2011	BAL	A	G	A	C
10	C34899–12	10/F	2012 Jan 20	NP	G	A	A	C
11	C63502–12	37/F	2012 Jan 23	BW	A/G	A	A	C

*ID, idenitifcation; SPT, sputum; NP, nasopharyngeal swab; BAL, bronchoavelor lavage; BW, bronchial washing.

In addition to macrolide resistance, molecular determinants of fluoroquinolone and tetracycline resistance in *M. pneumoniae* were examined. A previous report showed that substitutions at position 99 (83 for *Escherichia coli*) of *gyrA* and positions 81, 83, and 87 (78, 80, and 84 for *E. coli*) of *parC* were associated with fluoroquinolone resistance (*10*). In our study, none of the isolates contained any mutations that have been associated with fluoroquinolones resistance. Similarly, amplification and sequencing of 16S rRNA gene regions encompassing the tetracycline binding site did not show any mutations at positions 968 (T968C) and 1193 (G1193A), which have been shown to be associated with tetracycline resistance among *M. pneumoniae* (*11*).

Typing of *M. pneumonia*e isolates (42/96) by amplification and Sanger sequencing of almost the entire P1 adhesion gene was performed by using primer pairs ADH1/2, ADH3/4, and ADH2BF/R, which amplify 3 fragments of ≈2,280, 2,580 and 767 bp, respectively (*13*). Sequencing reactions were performed in both directions by using the Big Dye Terminator Cycle Sequencing Ready Reaction DNA Sequencing Kit in an ABI 3730 or 3750 automated sequencer (Applied Biosystems, Foster City, CA, USA).

P1 gene sequencing identified 6–11 variable number tandem repeat AGT sequences. Using sequence typing and comparing homology of nucleotide and amino acid sequences to reference sequences, we found that 16 (38%) isolates belonged to type 1 and shared high homology at nucleotide (99.7%–99.9%) and amino acid (99.5%–99.9%) levels. Seven of the P1 type 1 isolates had a point mutation in E179K, and 3 of these isolates also had a second mutation Q1232E compared with the type 1 reference strain (M129). Twenty-six (62%) isolates were characterized as type 2 and were differentiated within 3 variants (Table 2). Among 5 resistant isolates that were typed, 3 belonged to type 1 and 2 belonged to type 2.

**Table 2:** Typing of *Mycoplasma pneumoniae* isolates by P1 adhesion gene, Ontario, Canada, 2010–2011*

P1 gene type	No. isolates	Variant	Reference	GenBank accession no.†	Nucleotide homology, %	Amino acid homology, %	Nonsynonymous mutations compared with reference (no. isolates)	No. VTR AGT sequences	No. drug-resistant isolates
1	16	NA	M129	U00089.2	99.7–99.9	99.5–99.9	E179K (7), D559N (1), Q1232E (4)	7–11	3
2	8	NA	Mp1842	AF290002.1	99.6–99.9	99.5–99.8	S841L, E962D, I1302V	7–11	1
	3	2a	Mpn309	AP012303.1	99.8–99.9	99.5–99.6	Y210N (1), T837N (3)	6	0
	8	2b	T-103	AB691539.1	99.5–99.9	99.5–99.9	S1516P: 6 isolates had a deletion at 1244-QTNS-1247	7–9	1
	7	2c	P033	JN048891.1	99.8–100	99.7–100	V1411E	7–9	0

*VTR, variable tandem repeat. NA, not applicable (strains M129 and Mp1842 are considered prototypes representative for types 1 and 2). †The sequences of the P1 gene generated in this study have been deposited in GenBank (accession nos. KF154740–KF154759).

## Conclusions

In this study, 12.1% of *M. pneumoniae*–positive specimens contained mutations that are associated with macrolide resistance, and most (90.9%) specimens had a mutation at nt 2063. This finding is not surprising because this mutation has been shown to be predominant among macrolide-resistant *M. pneumoniae* and has been associated with high-level resistance (erythromycin MIC >64 mg/L) (*2**,**3**,**14*). None of the specimens contained any mutations at positions 2067 or 2617 because mutations at these positions are rare. Previous studies have shown reduced efficacy rate of macrolides for treating infections with *M. pneumoniae* isolates containing 3 mutations (*7**,**9*).

Typing of the P1 gene showed no clear association between macrolide-resistant isolates and specific subtype because 3 of the 5 resistant isolates belonged to type 1 and the remaining 2 belonged to type 2; there was no evidence of clonality. This finding is consistent with reports in which macrolide-resistant *M. pneumoniae* isolates of both types were found (*2**,**5**,**8*). However, according to a recent study in China, where macrolide-resistant *M. pneumoniae* is highly prevalent (>90%), most isolates were type 1 (*15*).

None of the *M. pneumoniae* isolates contained any *gyrA* gene mutations associated with fluoroquinolone resistance or any 16S rRNA gene mutations associated with tetracycline resistance. These findings are consistent with those of a study in which none of the isolates were resistant to fluoroquinolones or minocycline (*2*).
